# Chitosan-Based Drug Delivery System: Applications in Fish Biotechnology

**DOI:** 10.3390/polym12051177

**Published:** 2020-05-21

**Authors:** Yuanbing Wu, Ania Rashidpour, María Pilar Almajano, Isidoro Metón

**Affiliations:** 1 Secció de Bioquímica i Biologia Molecular, Departament de Bioquímica i Fisiologia, Facultat de Farmàcia i Ciències de l’Alimentació, Universitat de Barcelona, Joan XXIII 27–31, 08028 Barcelona, Spain; wuuanbing@gmail.com (Y.W.); aniyarashidpoor2017@gmail.com (A.R.); 2 Departament d’Enginyeria Química, Universitat Politècnica de Catalunya, Diagonal 647, 08028 Barcelona, Spain; m.pilar.almajano@upc.edu

**Keywords:** chitosan, gene delivery, gene overexpression, gene silencing, fish biotechnology

## Abstract

Chitosan is increasingly used for safe nucleic acid delivery in gene therapy studies, due to well-known properties such as bioadhesion, low toxicity, biodegradability and biocompatibility. Furthermore, chitosan derivatization can be easily performed to improve the solubility and stability of chitosan–nucleic acid polyplexes, and enhance efficient target cell drug delivery, cell uptake, intracellular endosomal escape, unpacking and nuclear import of expression plasmids. As in other fields, chitosan is a promising drug delivery vector with great potential for the fish farming industry. This review highlights state-of-the-art assays using chitosan-based methodologies for delivering nucleic acids into cells, and focuses attention on recent advances in chitosan-mediated gene delivery for fish biotechnology applications. The efficiency of chitosan for gene therapy studies in fish biotechnology is discussed in fields such as fish vaccination against bacterial and viral infection, control of gonadal development and gene overexpression and silencing for overcoming metabolic limitations, such as dependence on protein-rich diets and the low glucose tolerance of farmed fish. Finally, challenges and perspectives on the future developments of chitosan-based gene delivery in fish are also discussed.

## 1. Introduction

Chitosan is a cationic polymer of β (1-4)-linked 2-amino-2-deoxy-d-glucose interspersed by residual 2-acetamido-2-deoxy-β-d-glucose, derived from chitin by deacetylation under alkaline conditions. Chitin is the second most abundant polysaccharide in nature, after cellulose, and it is obtained from the external skeleton and skin of arthropods and insects. Chitin is also found in some microorganisms, yeast and fungi. Mucoadhesion, low toxicity, biodegradability and biocompatibility, as well as antioxidant, antibacterial, antifungal, antitumor and anti-inflammatory properties led, in recent years, to the increasing use of chitosan in a wide variety of pharmaceutical, biomedical and biotechnological fields, including wound healing, tissue engineering, bone regeneration, gene therapy, food industry and agriculture [1,2,3,4,5,6].

Chitosan has many desirable biological properties that make it a highly suitable carrier to deliver nucleic acids for the development of gene therapy assays. The goal of gene therapy is to introduce exogenous genetic material into target cells, with the aim of modifying the expression of specific genes. The efficient delivery of plasmid DNA to express exogenous genes or siRNA to knockdown the expression of target genes must overcome systemic and cell barriers, depending on the target tissue and nature of the molecular mechanism triggered by the gene therapy. Ideally, for safe nucleic acid delivery, the vector must establish a stable interaction with the cargo, protect it from the action of nucleases, reach target cells, enable crossing the cell membrane and, once inside the cell, facilitate escape from endosomes and lysosomes. Decomplexation from the carrier must allow plasmid DNA to cross the nuclear membrane and become transcribed, or in the case of siRNA, render the cargo in the cytosol [7,8,9].

Nucleic acid delivery into cells is facilitated by viral and non-viral vectors. The choice of the vector for gene therapy is a key step to properly reach target cells, confer protection from nucleases, cross the cell membrane, nucleic acid escape from endosomal vesicles, determine transient or permanent effects, allow transcription of delivered plasmid DNA and knockdown the expression of target genes by RNA interference (RNAi) [7,10].

Due to its high transfection efficiency, viral vectors are still used in most gene therapy assays. However, immunogenicity, acute inflammation and other unwanted effects, such as reversal of the wild-type phenotype associated with the use of viral vectors, have focused attention on the development of safer alternative gene delivery systems [9,11,12]. Non-viral vectors include lipid-based vectors and cationic polymers. Low transfection efficiency in vivo, reduced half-life of lipoplex circulation, cytotoxicity and other non-desired effects, such as complement activation, limit in vivo use of cationic lipids and lipid-based vectors [10,13,14,15,16]. Unlike viral vectors, cationic polymers, such as chitosan and its derivatives, exhibit increased ability to select target tissues, easy large-scale production, low toxicity and immunogenicity in vivo and biocompatibility [4,9,10]. In this review, we will summarize recent advances in chitosan-based formulations for delivering nucleic acids, and address current progress of the use of chitosan for fish biotechnology applications and gene therapy.

## 2. Chitosan as a Nucleic Acid Delivery Vector

**Figure 1:**
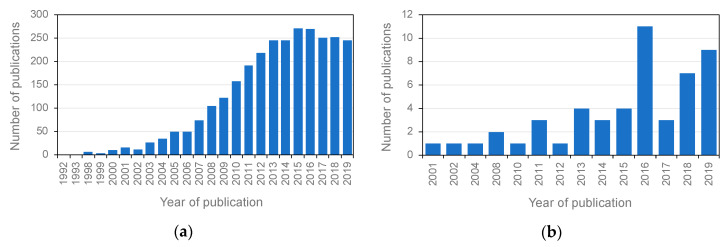
Web of Science (Clarivate Analytics) citations published until 2019 with the topics: (**a**) chitosan and gene therapy; (**b**) chitosan, fish and gene therapy.

The use of chitosan as a vector for nucleic acid delivery was proposed in 1995 [17]. A few years later, in 1998, in vivo administration of chitosan complexed with plasmid DNA to express a reporter gene in the upper small intestine and colon of rabbits was published [18]. It was in 2006 when chitosan nanoparticles encapsulating small interfering RNA (siRNA) were shown to be also effective for silencing the expression of target genes [19]. Since pioneering studies, much progress has been made in this area, and chitosan is considered, at present, one of the most effective non-viral gene delivery systems. Figure 1 shows Web of Science (Clarivate Analytics) citations, with the topics chitosan, fish and gene delivery until 2019.

The presence of numerous primary amine groups that are protonated at slightly acidic pH in chitosan allows electrostatic interaction with negatively charged nucleic acids. The stability of the complex formed between chitosan and nucleic acids allows oral, nasal, intravenous and intraperitoneal administration of chitosan–DNA complexes, and prevents dissociation before reaching the intracellular compartment [20,21,22]. Oral delivery would mainly result in intestinal absorption of the product [22]. Biodistribution of radioiodinated chitosan fractions with different molecular mass, intravenously injected to rats, showed rapid plasma clearance (<15% in the blood 5 min following treatment) and localization in the liver of most of the chitosan with diameter size >10 kDa (>50% at 5 min following intravenous administration and >80% at 60 min post-treatment). However, low molecular weight chitosan (<5 kDa) was cleared more slowly from the circulation and significantly less retained in the liver at the short- and long-term [20].

### 2.1. Chitosan Derivatization

Derivatization can greatly influence biodistribution of chitosan complexes. An illustrative example was developed by Kang et al. to down-regulate Akt2 expression for treatment of colorectal liver metastases in mice [23]. To protect siRNA from gastrointestinal degradation, facilitate active transport into enterocytes and enhance transportation to the liver through the enterohepatic circulation, the authors first obtained gold nanoparticles conjugated with thiolated siRNA (AR). The resulting complex was subsequently complexed with glycol chitosan−taurocholic acid (GT) through electrostatic interaction to generate AR-GT nanoparticles. Derivatization with taurocholic acid successfully protected Akt2-siRNA from gastrointestinal degradation and favored targeting to the liver through the enterohepatic circulation. Chitosan derivatization with hydrophilic ethylene glycol (glycol chitosan) increases solubility in water at a neutral/acidic pH. In addition, the reactive functional groups of glycol chitosan facilitate chemical modifications and formation of different derivatives useful for targeting gene delivery [24]. In addition to the properties of chitosan derivatives, the efficient delivery of the cargo greatly depends on chitosan polyplex properties, such as pH, molecular weight, deacetylation degree and N/P ratio [7,9].

The molecular weight of chitosan is a major factor affecting polyplex formation, the stability of the chitosan/DNA complex, cell entry, DNA unpacking after endosomal escape and transfection efficiency. Furthermore, the average particle size is highly dependent on the molecular weight of chitosan [7,9,25]. Chitosan between ~20–150 kDa forms chitosan–plasmid DNA complexes with diameter size of ~155–200 nm. High molecular weight chitosan >150 kDa losses solubility and favors aggregate formation, whereas chitosan of molecular weight <20 kDa tends to form polyplexes with diameter size >200 nm [26]. The optimal molecular weight range for stable chitosan–siRNA nanoparticle formation and efficient transfection and silencing effect is considered to be ~65–170 kDa [27].

**Figure 2:**
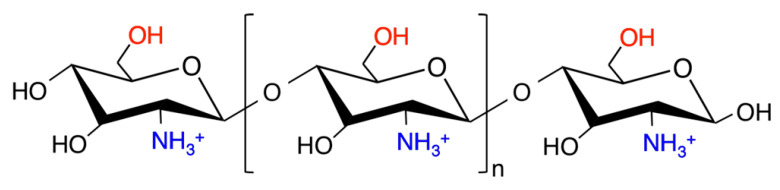
Schematic representation of chitosan. Functional groups C_2_-NH_2_ and C_6_-OH and are represented in blue and red color, respectively.

Chemical modification of chitosan can greatly improve desirable properties for gene delivery. Functional groups of chitosan include C_3_-OH, C_6_-OH, C_2_-NH_2_, acetyl amino and glycoside bonds [6,28]. Two of the functional groups, C_6_-OH and C_2_-NH_2_, have chemical properties that make them of particular interest for derivatization (Figure 2).

### 2.2. Chitosan Solubility

The water solubility of chitosan is low due to the presence of highly crystalline intermolecular and intramolecular hydrogen bonds, and can be greatly influenced by the pH, molecular weight and deacetylation degree [6,9,29]. The solubility of chitosan has been improved by introducing a hydrophilic group on amino or hydroxyl groups. Examples include: N-acylated chitosan derivatives, which exhibit enhanced biocompatibility, anticoagulability, blood compatibility and sustained drug release [6,30]; chitosan conjugation with saccharides through *N*-alkylation, such as glycosylation [3,31,32]; and the introduction of a quaternary ammonium salt group, which increases chargeability, mucoadhesion, crossing of mucus layers and binding to epithelial surfaces [6,33,34].

### 2.3. Stability of Chitosan Polyplexes

**Figure 3:**
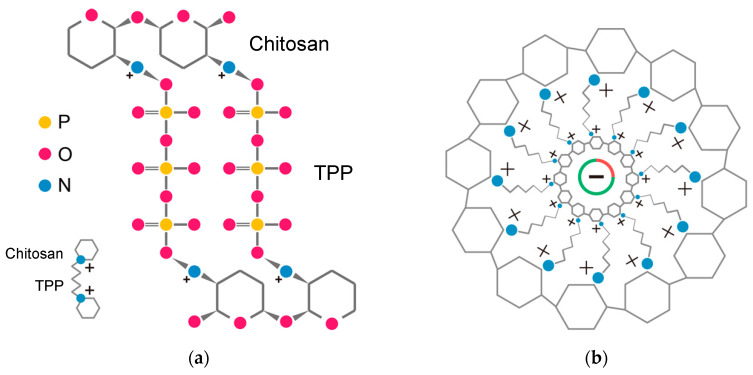
Molecular structure and electrostatic interactions of chitosan–tripolyphosphate (TPP) (**a**), and chitosan–TPP–plasmid DNA nanoparticles (**b**).

To increase the stability of chitosan-based formulations, a number of chitosan derivatives have been developed. Among them, PEGylation [35,36,37], glycosylation [3,38,39] and quaternization [39,40,41,42]. The choice of the method for preparing chitosan–nucleic acid complexes can also significantly affect stability of the complex and transfection efficiency. Katas and Alpar showed that for efficient siRNA-mediated silencing of the expression of target genes in CHO K1 and HEK 293 cells, nanoparticles produced by ionic gelation of tripolyphosphate (TPP) with chitosan were more efficient in delivering siRNA than chitosan–siRNA complexes and siRNA adsorbed onto chitosan–TPP nanoparticles. Chitosan–TPP-siRNA nanoparticles generated by ionic gelation presented higher binding capacity and loading efficiency [19]. During ionic gelation, TPP is a polyanion that crosslinks with positively charged chitosan through electrostatic interaction, avoiding the use of toxic reagents for chemical crosslinking, and allowing for the easy modulation of size and surface charge of the nanoparticles (Figure 3). The addition of TPP was shown to reduce the particle size and increase the stability of complexes in biological fluids [19,43,44,45,46,47]. The inclusion of hyaluronic acid in chitosan–siRNA polyplexes can be also a promising strategy to increase stability and targeting capacity, while lowering aggregation in the presence of serum proteins [48].

One major advantage of chitosan is that chitosan–DNA complexation protects DNA from DNase-mediated degradation, possibly as a result of modification of the DNA tertiary structure [20,49]. Cell penetration of chitosan-based gene delivery systems involves interaction between positively charged chitosan–nucleic acid polyplexes and negatively charged cell membrane components, such as heparan sulfate proteoglycans, enabling ATP-driven crossing of the cell membrane, or receptor-mediated endocytosis. In any case, chitosan polyplexes are internalized following the endocytic-lysosomal pathway [7].

### 2.4. Targeting Drug Delivery, Cellular Uptake and Intracellular Trafficking

Safe and effective therapies can be performed by using chitosan derivatives to improve target drug delivery. To this end, a variety of molecules can be conjugated to chitosan, such as proteins and peptides, polysaccharides, oligonucleotides and other molecules [4].

#### 2.4.1. Targeting Drug Delivery with Chitosan Derivatives

A common strategy to target drug delivery is based on ligand-receptor specificity. Cell-target delivery drugs can be thus enhanced by conjugation of chitosan–nucleic acid complexes with ligands that enable binding to receptors specifically found in the target cell membrane. Examples of ligands conjugated to chitosan formulations include transferrin, galactose and mannose. For instance, transferrin can be used as a targeting ligand for delivery into tumor cells through binding to the transferrin receptor, whose expression is enhanced in tumor cells to provide iron as a necessary cofactor for DNA synthesis and rapid cell proliferation [50,51,52]. The presence of asialoglycoprotein receptors on the hepatocyte surface and selective binding of asialoglycoprotein receptors to galactose allow galactosylated chitosan to target hepatocytes [53,54]. Mannosylated chitosan takes advantage of mannose recognition by mannose receptors to target dendritic cells [55].

Chitosan derivatives generally achieve mucosal adhesion through hydrogen bonding or non-specific, non-covalent, electrostatic interactions. Thiolated chitosan increases mucoadhesion and enhances crossing capability trough the cell membrane and ophthalmic drug delivery [56,57,58,59,60]. The mucoadhesive properties of chitosan derivatives allow oral administration and nasal immunization to treat respiratory diseases [61]. Other examples include O-carboxymethyl chitosan, which can be used for intestine-targeted drug delivery [62], and acetylated low molecular weight chitosan, for targeting the kidneys [63].

#### 2.4.2. Endosomal Escape, Unpacking and Nuclear Import of DNA

The proton sponge effect of chitosan gene delivery formulations allows endosomal escape before the maturation of early endosomes into late endosomes, and the ultimate fusion with lysosomes. The increasing acidification in early endosomes generated by the V-type ATPase proton pump results in progressive protonation of the amine groups of chitosan (pKa value of ~6.5), leading to the influx of water and chloride ions into the endosomes, increased osmotic swelling, endosome lysis and cytosolic release of the endosomal content [9,64]. The endosomal release of chitosan polyplexes can be enhanced by fusogenic peptides [65,66] and pH-sensitive neutral lipids [67]. Efficient transfection and endosomal escape of chitosan polyplexes can be also enhanced by chitosan–polyethylenimine (PEI) copolymeric delivery systems. PEI is a cationic polymer non-viral vector with high transfection efficiency and a strong buffering capacity, which may enhance the influx of chloride anions, osmotic swelling and endosomal lysis. However, PEI-dependent cytotoxic effects constitute a major concern when using PEI for gene delivery [7,68,69,70]. In contrast, chitosan–PEI complexes exhibit efficient uptake by target cells, high transfection efficiency and negligible toxicity [36,71,72,73,74,75].

Following endosomal escape into the cytosol, chitosan polyplexes carrying DNA must be unpacked, and the entrance of loaded DNA into the nucleus is needed for transfection. The molecular events that mediate DNA unpacking after endosomal release and translocation to the nucleus remain not fully understood. It is generally accepted that, in non-dividing cells, molecules smaller than ∼40 kDa can passively diffuse through the nuclear pores, while larger molecules must carry nuclear localization signals for active transportation [68]. Sun et al. largely improved DNA unpacking from chitosan and transfection efficiency upon the conjugation of chitosan with small peptides that can be phosphorylated [76]. The phosphorylation of conjugated peptides mimics the process leading to genomic DNA release and the activation of transcription, mediated by histone phosphorylation. In addition, the introduction of negatively charged phosphate groups may result in electric repulsion between DNA and chitosan conjugated with phosphorylated peptides. Hence, further enhancement of transfection was obtained by conjugating chitosan with small peptides carrying a nuclear localization signal, in addition to a potentially phosphorylatable serine residue [77]. Exogenous gene expression was improved through a mechanism that enabled DNA import into the nucleus, and enhanced unpacking by the action of nuclear histone kinases. Miao et al. improved endosomal escape and intracellular drug release in HepG2.2.15 cells by loading DNA into a redox-responsive chitosan oligosaccharide-SS-octadecylamine (CSSO) polymer. Intracellular reduction and cleavage of CSSO disulfide bonds ‘–SS-’ by gluthation allowed rapid DNA release [78].

For strategies aiming RNAi on target genes, chitosan has been mostly complexed with siRNA, microRNA (miRNA) and plasmids expressing short hairpin RNA (shRNA). After unpacking, siRNA/miRNA associates with RNA-induced silencing complex (RISC) in the cytosol. The RNAi-guided complex hybridizes with target mRNA, leading to mRNA cleavage and/or translation repression, and subsequent inhibition of protein synthesis [9,10,48,79]. The use of shRNA expression plasmids allowing long lasting expression of siRNA may improve RNAi in vivo. Following plasmid DNA transcription in the nucleus, the transcribed shRNA is processed by Drosha, exported to the cytosol and processed by Dicer, leading to cleavage of double-stranded shRNA and the formation of specific siRNA [75,80,81,82,83,84,85].

**Figure 4:**
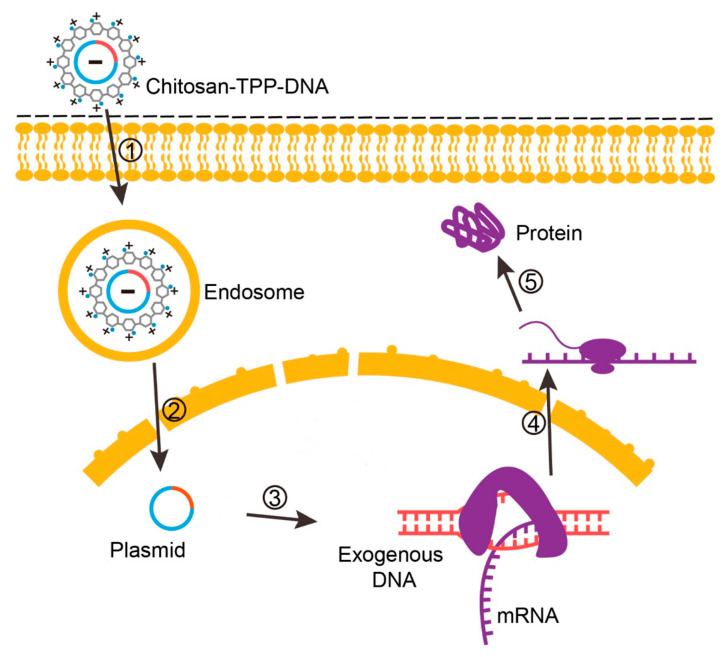
Cellular events associated with chitosan-based plasmid delivery for exogenous gene expression. 1, Cellular uptake of chitosan–DNA by endocytosis. 2, Endosomal escape of the chitosan–DNA complex, plasmid dissociation from chitosan and translocation to the nucleus. 3, Transcription of plasmid (exogenous DNA) in the nucleus and mRNA generation. 4, Translation of newly transcribed mRNA in the cytosol. 5, Exogenous protein assembly.

**Figure 5:**
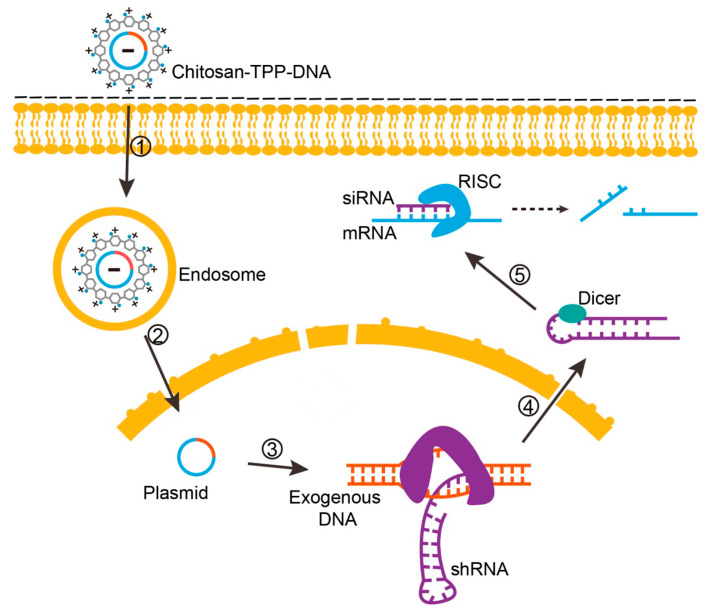
Cellular events associated with chitosan-based plasmid delivery for short hairpin RNA (shRNA) expression, siRNA formation and target gene silencing. 1, Cellular uptake of chitosan–DNA by endocytosis. 2, Endosomal escape of chitosan–DNA complex, plasmid dissociation from chitosan and translocation to the nucleus. 3, Transcription of plasmid (exogenous DNA) in the nucleus and generation of shRNA. 4, Transportation of shRNA to the cytosol and association with Dicer to generate siRNA. 5, siRNA association with RNA-induced silencing complex (RISC) and target mRNA by base pairing, resulting in mRNA cleavage and/or translation repression, and subsequent inhibition of protein synthesis.

**Figure 6:**
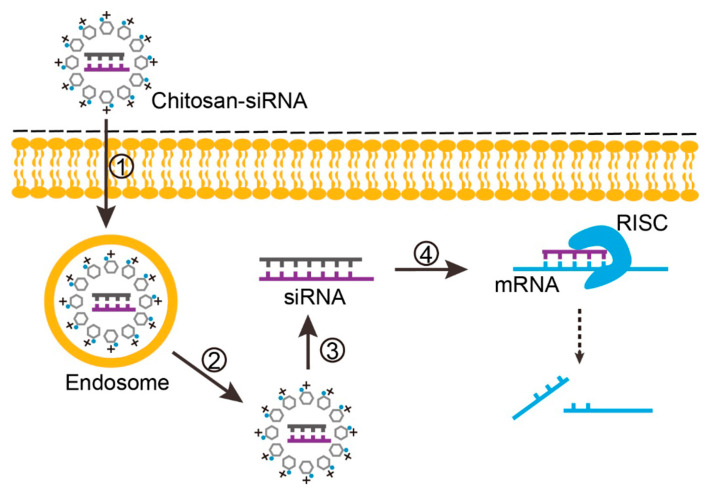
Cellular events associated with chitosan-based siRNA delivery for target gene silencing. 1, Cellular uptake of chitosan–siRNA by endocytosis. 2, Endosomal escape of chitosan–siRNA. 3, Dissociation of siRNA from chitosan. 4, siRNA association with RISC and target mRNA by base pairing, resulting in target mRNA cleavage and/or translation repression, and subsequent inhibition of protein synthesis.

Sequential events associated with three illustrative examples using chitosan to deliver nucleic acids are represented in Figure 4 (chitosan–TPP complexed with a plasmid construct, to express an exogenous protein), Figure 5 (chitosan–TPP complexed with a plasmid construct, to express a shRNA designed for target gene silencing) and Figure 6 (chitosan loading siRNA for target gene silencing).

## 3. Use of Chitosan in Fish Biotechnology

Chitosan and its derivatives are widely used in aquaculture. Low toxicity, biodegradability, biocompatibility, bioadhesion and immunomodulatory properties make chitosan and its derivatives of increasing interest for the fish farming industry as dietary additives, non-viral vectors enabling fish vaccination and protection against diseases, control of gonadal development and for the gene therapy-based modulation of fish metabolism.

### 3.1. Chitosan and Its Derivatives as Dietary Additives

Dietary supplementation with chitosan and its derivatives has been shown to improve fish growth performance, non-specific immunity and antioxidant effects [86,87]. However, the strategy for chitosan dietary supplementation in fish requires extensive investigation, according to the species and the growth stage of fish.

#### 3.1.1. Dietary Supplementation with Chitosan

The inclusion of chitosan as feed additive for fish has been receiving attention since the 1980s [88]. Shiau et al. reported that inclusion of dietary levels of chitosan from 2% to 10% for 28 days decreases the weight gain and increases the feed conversion ratio (FCR) in hybrid tilapia (*Oreochromis niloticus* × *Oreochromis aureus*) [89]. However, other studies performed in *Oreochromis niloticus* showed positive effects of chitosan on fish growth. Feed supplementation of tilapia with chitosan (0–8 g/kg dry diet) for 56 days led to the conclusion that 4 g/kg of chitosan was the optimal dose to promote the highest body weight gain (BWG) rate and specific growth rate (SGR) [90]. Similarly, chitosan supplementation at 5 g/kg diet for 60 days improved growth performance, BWG, SGR and FCR in tilapia [91]. The contradictory effects reported for chitosan on tilapia growth could be attributed to the fact that the studies were performed using different fish growth stages. Indeed, the initial weight of fish in the study by Shiau et al. was of 0.99 ± 0.01 g, while the latter two reports used a significantly higher initial body weight (50.1 ± 4.1 g and 39.3 ± 0.3 g, respectively).

In addition to the developmental stage and amount of dietary chitosan supplied, chitosan effects exerted on fish growth performance also seem to depend on the species [87]. According to the effect observed on SGR, the apparent digestibility coefficient of dry matter and the apparent digestibility coefficient of protein, 75 days of feeding on diets supplemented with 10–20 g chitosan/kg significantly reduced the growth performance of gibel carp (*Carassius gibelio*) (initial body weight, 4.80 ± 0.01 g) [92]. However, the supply of 0–0.2 g chitosan/kg diet caused a dose dependent increase of the average daily weight and SGR in post-larvae sea bass (*Dicentrarchus labrax*) [93]. Yan et al. also reported that dietary supplementation of 0%–5% chitosan improved growth performance by inducing dose dependent increases of BWG and SGR, while FCR decreased [94]. Similarly, 70 days of supplementation with 1–5 g chitosan/kg diet of loach fish (*Misgurnus anguillicadatus*) with an average body weight of 3.14 ± 0.05 g, significantly increased BWG, SGR and condition factor (CF), whereas it decreased FCR [95]. In contrast, Najafabad et al. found that Caspian kutum (*Rutilus kutum*) fingerlings (1.7 ± 0.15 g) supplied with 0–2 g chitosan/kg diet for 60 days showed no effect of final weight, SGR and condition factor [96].

The positive effect of chitosan on the growth performance of some fish species might result from its role in nonspecific immunity. Chitosan acts as an immunostimulary drug through induction of nonspecific immunity in fish. In loach fish, the dietary supplement of chitosan increased the serum levels of factors considered as immune boosters, such as the content of immunoglobulin M (IgM), complement component 3 (C3) levels, the activity of lysozyme, acid phosphatase and alkaline phosphatase, as well as increased the survival rate after being challenged by *Aeromonas hydrophila* [95]. In accordance with the immune boost, other investigations also showed immune reinforcement by chitosan, when fish were challenged by bacteria in regard to immunoglobulin content, serum lysozyme, bactericidal activity, immune-related gene expression, phagocytosis and respiratory burst activity [90,92,94,97]. Consistently, chitosan was shown to modify hematological parameters of fish, which are also considered important indicators of immunostimulation. In Asian seabass (*Lates calcarifer*), chitosan supplement during 60 days at 5–20 g/kg diet increased red blood cells (RBC), white blood cells (WBC), total serum protein, albumin and globulin [98]. Supplementation with chitosan was reported also to increase RBC, WBC, haemoglobin, lymphocytes, monocytes, neutrophils and thrombocytes in mrigal carp (*Cirrhinus mrigala*) and kelp grouper (*Epinephelus bruneus*) [99,100,101].

Concomitant to the effects on immunity, chitosan also elevates antioxidant responses in fish. In loach fish, the activity of phenoloxidase, superoxide dismutase (SOD) and glutathione peroxidase (GPx) increased after 12 weeks of chitosan supplementation [95]. Similarly, chitosan induced the activity of SOD and catalase (CAT) after 56 days of dietary supplementation in tilapia [90], and the mRNA levels of SOD, CAT, GPx and nuclear factor erythroid 2-related factor 2 [94]. The protective effect of chitosan from oxidative stress was also reported in olive flounder (*Paralichthys olivaceus*) challenged with H_2_O_2_ [97]. The authors observed that chitosan-coated diets significantly narrowed the increase of protein carbonyl formation and DNA damage in the plasma.

#### 3.1.2. Dietary Supplementation with Chitosan Nanoparticles

Wang et al. reported that BWG significantly increased in tilapia (initial body weight, 23.6 ± 1.2 g) fed with chitosan nanoparticles (5 g/kg dry diet) [102]. Similar results were described by other authors. Chitosan nanoparticle intake increased final weight, weight gain, SGR and FCR in tilapia supplied for 45 days with 0–2 g/kg (initial body weight, 19.8 ± 0.6 g) and 70 days for 1–5 g/kg (initial body weight, 5.66 ± 0.02 g). In these reports, innate immunity was also enhanced and fish exhibited increased respiratory burst activity, lysozyme malondialdehyde, CAT and SOD activity, and hematological parameters such as RBC, hematocrit, hemoglobin, mean corpuscular volume, WBC and platelets [103,104]. Remarkably, optimal supplement of dietary chitosan nanoparticles to improve growth and immunity against pathogens may vary, according to parameters such as developmental growth stage and species.

Dietary supplementation of chitosan nanoparticles complexed with vitamin C and thymol is more effective in enhancing immunity than supplementation with the single additives. Dietary chitosan–vitamin C nanoparticles slightly improved growth performance of tilapia, while inducing the viscerosomatic index, therefore decreasing economic performance. However, when fish fed chitosan–vitamin C nanoparticles were challenged by imidacloprid-polluted water, chitosan–vitamin C supplementation significantly strengthened immunity and antioxidant activity, including the activity of lysozyme, glutathione reductase and CAT, C3 and immunoglobulins [105]. Growth effects of dietary supplementation with chitosan nanoparticles mixed with thymol, the most important phenolic compound in *Thymus vulgaris* essential oil, were evaluated on hematological parameters, and the liver and kidney function in tilapia [106]. The results showed that chitosan–thymol nanoparticle supplementation increased feed efficiency and protein efficiency ratio, while it had moderated effects on final weight, weight gain and SGR. Nevertheless, chitosan–thymol produced a synergistic effect on lymphocytes and monocyte leukocytes. The use of chitosan nanoparticles as feed additive is limited by the fact that it can exhibit toxic effects at high levels. In this regard, chitosan nanoparticles significantly decreased hatching rate and survival rate of zebrafish (*Danio rerio*) when the immersion concentration reached 20 and 30 μg/mL or higher [107,108].

#### 3.1.3. Dietary Supplementation with Chitin and Chitooligosaccharide

Meanwhile the inclusion of chitin in the diet has no significant effects on fish growth performance [109,110,111], chitooligosaccharide (COS) enhances growth performance parameters such as BWG, hepatosomatic and intestosomatic index, SGR and FCR in a number of fish species, including juvenile largemouth bass (*Micropterus salmoides*) [112], striped catfish (*Pangasianodon hypophthalmus*) [113], Nile tilapia (*Oreochromis niloticus*) [114], tiger puffer (*Takifugu rubripes*) [115], koi (*Cyprinus carpio koi*) [116], and silverfish (*Trachinotus ovatus*) [117]. Similarly as in most fish species, dietary supplementation with low molecular weight and highly deacetylated COS enhances growth performance, innate immunity and digestive enzyme activity in Pacific white shrimp (*Litopenaeus vannamei*) [118]. However, the effect of dietary COS may depend on the species. In this regard, dietary COS supplementation was reported to cause not significant effects on weight gain, FCR and the survival rate in hybrid tilapia (*Oreochromis niloticus×O. aureus*) [109]. Similar results were reported for rainbow trout (*Oncorhynchus mykiss*) [119]. Incomplete intestinal development in early developmental stages may contribute to the lack of COS effect on growth performance observed in several fish species.

A number of studies showed that both chitin and COS can be potentially utilized as immunostimulants in fish. Respiratory burst activity, phagocytic activity and lysozyme activity, which are considered indicators of non-specific immunity, have been shown to be significantly stimulated by chitin and COS in a number of fish species, including juvenile largemouth bass (*Micropterus salmoides*) [112], Nile tilapia (*Oreochromis niloticus*) [114], striped catfish (*Pangasianodon hypophthalmus*) [113] and mrigal carp (*Cirrhina mrigala*) [99]. Chitin and COS also induce other immunity parameters, such as nitric oxide production, inducible nitric oxide synthase (iNOS) activity and gene expression [112,120], leukocyte count [99,112,116] and complement activity [99,100].

### 3.2. Chitosan as a Carrier for Drug Delivery in Fish

Chitosan is nanoscale, biodegradable, biocompatible, hemocompatible, simple and mild for preparation conditions, and is highly efficient for drug loading. Therefore, chitosan has been used for loading a variety of bioactive compounds, such as vitamins, metal ions, inactivated pathogens for vaccines, proteins and nucleic acids in a variety of applications in fish farming. In addition, loading into chitosan can significantly boost the bioeffects of these compounds.

#### 3.2.1. Chitosan Loading Chemical Compounds

The sustained release of compounds complexed with chitosan nanoparticles fulfills the requirements of artificial breeding in fish farming and enable delivery and cell uptake of compounds with low toxicity [121,122]. Chitosan nanoparticles loaded with vitamin C, an important but labile antioxidant, were proven to enhance sustained vitamin C release in the stomach, the intestine and in serum after oral administration in rainbow trout (*Oncorhynchus mykiss*) [123]. Chitosan–vitamin C nanoparticles exhibited a markedly high antioxidant activity and no toxicity up to 2.5 mg/mL in the culture medium of ZFL cells, a zebrafish liver-derived cell line. In addition, chitosan–vitamin C nanoparticles showed the capability to penetrate the intestinal epithelium of *Solea senegalensis* [124]. Several studies evaluated chitosan nanoparticles loading aromatase inhibitors and eurycomanone, compounds that promote gonadal development. Chitosan-mediated delivery of aromatase inhibitors and eurycomanone prolonged serum presence, improved testicular development with lack of testicular toxicity, and led to higher serum concentrations of reproductive hormones [125,126,127,128].

#### 3.2.2. Chitosan Loading Metal Ions

Loading with chitosan facilitates delivery of metal ions that are micronutrients and antibacterial factors, such as selenium and silver, to fish in culture. Barakat et al. showed that chitosan–silver nanoparticles successfully treated European sea bass larvae infected with *Vibrio anguillarum*. Chitosan–silver nanoparticles significantly decreased the bacterial number and improved fish survival [129]. In addition, dietary supplementation with chitosan–silver nanoparticles were shown to altering gut morphometry and microbiota in zebrafish. Feeding with chitosan–silver nanoparticles increased *Fusobacteria* and *Bacteroidetes* phyla, goblet cell density and villi height, while upregulated the expression of immune-related genes [130]. Similarly, chitosan–selenium nanoparticles had immunostimulary roles and increased disease resistance in zebrafish and *Paramisgurnus dabryanus* by improving the activity of lysozyme, acid phosphatase and alkaline phosphatase, phagocytic respiratory burst and splenocyte-responses towards concanavalin A [131,132].

#### 3.2.3. Chitosan Loading Inactivated Pathogens

Vaccines against pathogens is a major challenge in aquaculture. In this regard, chitosan can be used as proper carrier and adjuvant to enhance effectiveness of vaccination. A number of inactivated bacteria and virus have been evaluated with chitosan or its derivatives as adjuvant against infections in fish. Vaccines, such as inactivated *Edwardsiella ictaluri* and infectious spleen and kidney necrosis virus, have been tested with chitosan in yellow catfish (*Pelteobagrus fulvidraco*) and Chinese perch (*Siniperca chuasi*), respectively. Chitosan enhanced incorporation into the host cells and improved fish survival rate and immune response, increasing IgM content, lysozyme activity and mRNA levels of interleukin (IL)-1β, IL-2 and interferon (IFN)-γ2 [133,134]. A mixture of COS and inactivated *Vibrio anguillarum* vaccine significantly reduced zebrafish mortality against *Vibro anguillarum* [135], while COS combined with inactivated *Vibrio harveyi* also markedly increased survival rate, IgM and the expression of immune-related genes, such as IL-1β, IL-16, tumor necrosis factor-alpha (TNF-α) and major histocompatibility complex class I alpha (MHC-Iα), in the grouper ♀*Epinephelus fuscoguttatus*×♂*Epinephelus lanceolatus* [136]. Similarly, rainbow trout (*Oncorhynchus mykiss*) immunized against bacterial infection (*Lactococcus garvieae* and *Streptococcus iniae*) through chitosan–alginate coated vaccination exhibited a higher survival rate, immune-related gene expression, and antibody titer than fish submitted to non-coated vaccination [137].

Olive flounder (*Paralichthys olivaceus*) vaccinated against inactivated viral haemorrhagic septicaemia virus encapsulated with chitosan through oral and immersion routes showed effective immunization in the head kidney, which is considered as the primary organ responsible for the initiation of adaptive immunity in fish, skin and intestine, which are regarded as the main sites for antigen uptake and mucosal immunity. Additionally to upregulation of IgM, immunoglobulin T (IgT), polymeric Ig receptor (pIgR), MHC-I, major histocompatibility complex class II (MHC-II) and IFN-γ in the three tissues, caspase 3 was also highly induced 48 h post-challenge, suggesting cytotoxicity due to rapid T-cell response and impairment of viral proliferation [138].

Coating chitosan with membrane vesicles from pathogens such as *Piscirickettsia salmonis* was also shown to be an effective strategy to induce immune response in zebrafish (*Danio rerio*) and upregulation of CD 4, CD 8, MHC-I, macrophage-expressed 1, tandem duplicate 1 (Mpeg1.1), TNFα, IL-1β, IL-10, and IL-6 [139].

#### 3.2.4. Chitosan Loading Proteins

Effectiveness of fish vaccination against infections can be also improved with antigenic proteins derived from bacteria and virus. For example, chitosan nanoparticles encapsulated with the recombinant outer membrane protein A of *Edwardsiella tarda* was used for oral vaccination of fringed-lipped peninsula carp (*Labeo fimbriatus*). Treated fish showed significant higher levels of post-vaccination antibody in circulation and survival rate against *Edwardsiella tarda* [140]. In another study, oral vaccination with alginate-chitosan microspheres encapsulating the recombinant protein serine-rich repeat (rSrr) of *Streptococcus iniae* were evaluated and the results showed that lysozyme activity and immune-related genes were induced, leading to a 60% increased survival rate of channel catfish (*Ictalurus punctatus*) against *Streptococcus iniae* infection [141]. In grass carp (*Ctenopharyngodon idella*), chitosan was also used for carrying the immunomodulatory factor IFN-γ2. Treatment with chitosan–*Ctenopharyngodon idella* IFN-γ2 highly upregulated inflammatory factors, leading to severe inflammatory damage in the intestine, hepatopancreas and decreased survival rate [142].

#### 3.2.5. Chitosan Loading Nucleic Acids

Compared to chitosan-based gene delivery in other organisms, gene therapy methodologies using chitosan for improving desirable traits in farmed fish have great potential for development (Figure 1b). A number of studies addressed the characterization of factors that can influence the efficiency of chitosan loading and nucleic acid release, such as the average diameter, zeta potential and encapsulation efficiency of chitosan–DNA microspheres or nanospheres. Table 1 summarizes chitosan–plasmid DNA encapsulation efficiency and changes in particle diameter and zeta potential before and after encapsulation for fish biotechnology studies. Existing data show that the diameter of chitosan nanospheres before loading DNA mostly ranged from ~30 to ~230 nm, while encapsulation with plasmid DNA led to ~40–190 nm diameter increase. The zeta potential indicates the surface charge on the particles. A higher positive zeta potential suggests higher stability of nanoparticles in the suspension [143]. The zeta potential before loading plasmid DNA were ~25–33 mV, which mostly tended to decrease to ~14–18 mV. The exception was reported by Rather et al., who found that zeta potential of chitosan nanospheres increased ~6 mV following DNA encapsulation [144]. DNA encapsulation efficiency was generally higher than 80%, which indicates that chitosan is capable to load a high mass of DNA, which in turn may benefit many applications in aquaculture.

Chitosan-encapsulated DNA is more stable in vivo, exhibit sustained-release and increased cell uptake than naked DNA. Taken together, these factors confer chitosan-delivered DNA a particular expression profile regarding tissue distribution, persistence of expression and abundance in fish. Sáez et al. found that intramuscular injection led to a restricted expression to adjacent tissues of both chitosan-encapsulated DNA and naked DNA, while the oral administration of chitosan-encapsulated DNA, largely used for fish vaccination studies, showed enhanced expression not only in the intestine, but also in the liver of gilthead sea bream (*Sparus aurata*) [152,155]. Furthermore, oral administration of chitosan nanoparticles loaded with pCMVβ, a plasmid encoding for *Escherichia coli* β-galactosidase, enabled sustained detection of the exogenous plasmid and bacterial β-galactosidase activity in the liver and the intestine of *Sparus aurata* juveniles up to 60 days posttreatment [152].

Through the immersion route, Rao et al. showed that chitosan-coated DNA was confined to the surface area of rohu (*Labeo rohita*), i.e., gill, intestine and skin-muscle, while no detection was observed in the kidney and the liver. Naked DNA was undetectable due to degradation [158]. Oral delivery seems to have a wider distribution of chitosan-encapsulated DNA, being found in the stomach, spleen, intestine, gill, muscle, liver, heart and kidney [148,154,159]. Chitosan-encapsulated DNA has longer and more abundant presence than naked DNA after administration. For example, Rajesh Kumar et al. showed that antibody in serum from fish immunized with a chitosan–DNA vaccine was 30% higher than naked DNA after 21 days of oral immunity [160]. The presence of DNA vaccine was reported more than 90 days after oral administration of chitosan–DNA [145]. Additionally, Rather et al. reported that chitosan–DNA induced 2-fold longer and higher peak abundant expression of downstream genes than naked DNA [144].

### 3.3. Chitosan-Based Applications in Fish Biotechnology and Gene Therapy

In recent years, chitosan has been increasingly used for drug and gene delivery in fish biotechnology. Most of the studies used chitosan-based systems to improve oral vaccination, control of gonadal development, and the modification of fish intermediary metabolism.

#### 3.3.1. Fish Vaccination

DNA vaccines delivered by chitosan significantly increase relative percent survival of fish at a range of 45%–82% against bacterial and viral infection [151,156]. Higher doses of chitosan–DNA vaccines resulted in concomitant increase of fish relative percent survival from ~47% to ~70% [154]. In addition, DNA vaccination with chitosan stimulated expression of immune-related genes. Zheng et al. reported upregulation of the expression of immune-related genes, such as interferon-induced GTP-binding protein Mx2 (MX2), IFN, chemokine receptor (CXCR), T-cell receptor (TCR), MHC-Iα and MHC-IIα, 7 days after oral vaccination against reddish body iridovirus in turbot (*Scophthalmus maximus*). A 10-fold higher expression of TNF-α gene expression was found in the hindgut [149].

In addition to the short-term modification of the expression levels of immune-related genes, the administration of chitosan–DNA vaccines also promote a sustained effect after treatment. Valero et al. found that European sea bass (*Dicentrarchus labrax*) orally vaccinated with chitosan-encapsulated DNA against nodavirus failed to induce circulating IgM. However, the expression of genes involved in cell-mediated cytotoxicity (TCRβ and CD8α) and the interferon pathway (IFN, MX and IFN-γ) were upregulated. Three months following vaccination, challenged fish exhibited partial protection with retarded onset of fish death and lower cumulative mortality [151]. Kole et al. immunized rohu (*Labeo rohita*) with chitosan nanoparticles complexed with a bicistronic DNA plasmid encoding the antigen *Edwardsiella tarda* glyceraldehyde 3-phosphate dehydrogenase and the immune adjuvant gene *Labeo rohita* IFN-γ [156]. Follow-up of the expression of immune-related genes in the the kidney, liver and spleen showed maximal upregulation of IgHC (IgM heavy chain), iNOS, toll like receptor 22 (TLR22), nucleotide binding and oligomerization domain-1 (NOD1) and IL-1β at 14 days post immunization. The authors also confirmed that oral and immersion vaccination with chitosan–DNA nanoparticles enhances the fish immune response to a greater extent than intramuscular injection of naked DNA. In another study, the oral vaccination of rainbow trout fry with chitosan–TPP nanoparticles complexed with pcDNA3.1-VP2, showed that the expression of genes related with innate immune response, IFN-1 and MX, reached maximal values at 3 days postvaccination and 7 days after boosting (22 days postvaccination), while, with regard to genes involved in the adaptative immune response, CD4 peaked at 15 days postvaccination and IgM and IgT at 30 days postvaccination [154].

#### 3.3.2. Control of Gonadal Development

Chitosan nanoparticles have been used for drug delivery in studies aiming proper gonadal development in fish farming. Bhat et al. administered chitosan conjugated with salmon luteinizing hormone-releasing hormone (sLHRH) into walking catfish (*Clarias batrachus*) to promote gonadal development. Chitosan-conjugated sLHRH and naked sLHRH exerted similar effects: upregulation of Sox9 expression in the gonads and increase of circulating steroid hormonal levels, testosterone and 11-ketotestosterone in males and testosterone and 17β-estradiol in females. However, sLHRH conjugation with chitosan induced sustained and controlled release of the hormones with maximal levels observed in the last sampling point of the experiment (36 h posttreatment), while naked sLHRH peaked circulating steroid hormones at 12 h posttreatment [150]. Similarly, compared to the administration of naked kisspeptin-10, intramuscular injection of chitosan-encapsulated kisspeptin-10 in immature female *Catla catla* caused a delayed but greater increase of gonadotropin-releasing hormone, luteinizing hormone and follicle-stimulating hormone expression, as well as circulating levels of 11-ketotestosterone and 17β-estradiol [144].

With the ultimate goal of controlling gonadal development in fish, chitosan was also assayed for gene delivery. In walking catfish (*Clarias batrachus*), intramuscular administration of chitosan nanoparticles conjugated with an expression plasmid encoding steroidogenic acute regulatory protein (StAR), a major regulator of steroidogenesis, also resulted in long-lasting stimulatory effects than administration of the naked plasmid construct on the expression of key genes in reproduction, cytochrome P450 (CYP) 11A1, CYP17A1, CYP19A1, 3β-hydroxysteroid dehydrogenase and 173β-hydroxysteroid dehydrogenase [153].

#### 3.3.3. Control of Fish Metabolism

Chitosan has been used for enhancing fish digestibility, the absorption of food constituents and increasing the utilization of dietary carbohydrate in carnivorous fish. To supplement exogenous proteolytic enzymes and thus facilitate protein digestion and amino acid absorption, Kumari et al. orally administered chitosan–TPP nanoparticles encapsulating trypsin to *Labeo rohita* over 45 days. Treatment with chitosan–TPP–trypsin enhanced nutrient digestibility, intestinal protease activity and transamination activity, alanine aminotransferase (ALT) and aspartate aminotransferase (AST), in the liver and the muscle [161].

The substitution of dietary protein by cheaper nutrients with reduced environmental impact in farmed fish is a challenging trend for sustainable aquaculture [162]. However, the metabolic features of fish, particularly carnivorous fish, constrain the replacement of dietary protein by other nutrients in aquafeeds. Carnivorous fish exhibit a preferential use of amino acids as fuel and gluconeogenic substrates, and thus require high levels of dietary protein for optimal growth. Instead, carbohydrates are metabolized markedly slower than in mammals, and give rise to prolonged hyperglycemia [163,164]. The essential role of the liver in controlling the intermediary metabolism makes this organ an ideal target for investigating and modifying the glucose tolerance of farmed fish.

To overcome metabolic limitations of carnivorous fish, in recent years we synthesized chitosan–TPP nanoparticles, complexed with plasmid DNA, to induce in vivo transient overexpression and the silencing of target genes in the liver of gilthead sea bream (*Sparus aurata*). With the aim of decreasing the use of amino acids for gluconeogenic purposes and improving carbohydrate metabolism in the liver, chitosan–TPP nanoparticles complexed with a plasmid overexpressing a shRNA designed to silence the expression of cytosolic ALT (cALT) were intraperitoneally administered to *Sparus aurata* juveniles. Seventy-two hours posttreatment, a significant decrease in cALT1 mRNA levels, immunodetectable ALT and ALT activity was observed in the liver of treated fish. Knockdown of cALT expression to ~63%–70% of the values observed in control fish significantly increased the hepatic activity of key enzymes in glycolysis, 6-phosphofructo 1-kinase (PFK1) and pyruvate kinase, and protein metabolism, glutamate dehydrogenase (GDH). In addition to showing efficient gene silencing after administration of chitosan–TPP–DNA nanoparticles, the findings supported evidence that the downregulation of liver transamination increased the use of dietary carbohydrates to obtain energy, and thus made it possible to spare protein in carnivorous fish [80].

Following the same methodology, we showed that the shRNA-mediated knockdown of GDH significantly decreased GDH mRNA and immunodetectable levels in the liver, which, in turn, reduced GDH activity to ~53%. Downregulation of GDH decreased liver glutamate, glutamine and 2-oxoglutarate, as well as the hepatic activity of AST, while it increased 2-oxoglutarate dehydrogenase activity and the PFK1/fructose-1,6-bisphosphatase (FBP1) activity ratio. Therefore, by reducing hepatic transdeamination and gluconeogenesis, the knockdown of GDH could impair the use of amino acids as gluconeogenic substrates and facilitate the metabolic use of dietary carbohydrates [81].

**Figure 7:**
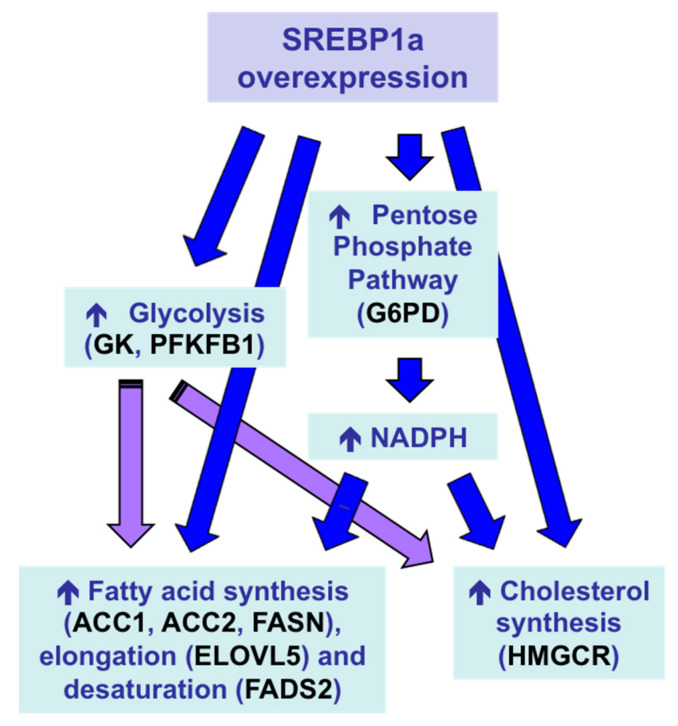
Multigenic action and metabolic effects in the liver of *Sparus aurata* after intraperitoneal administration of chitosan–TPP–DNA nanoparticles to overexpress exogenous SREBP1a [157]. ACC1, acetyl-CoA carboxylase 1; ACC2, acetyl-CoA carboxylase 2; ELOVL5, elongation of very long chain fatty acids protein 5; FADS2, fatty acid desaturase 2; G6PD, glucose 6-phophate dehydrogenase; GK, glucokinase; HMGCR, 3-hydroxy-3-methylglutaryl-coenzyme A reductase; PFKFB1, 6-phosphofructo 2-kinase/fructose 2,6-bisphosphatase.

With the aim of inducing a multigenic action leading to a stronger protein-sparing effect, *Sparus aurata* were intraperitoneally injected with chitosan–TPP nanoparticles complexed with a plasmid expressing the N-terminal nuclear fragment of hamster SREBP1a, a transcription factor that—in addition to exhibiting strong transactivating capacity of genes required for fatty acid, triglycerides and cholesterol synthesis—previous reports showed can also transactivate the promoter of genes encoding key enzymes in hepatic glycolysis, glucokinase (GK) and 6-phosphofructo 2-kinase/fructose 2,6-bisphosphatase (PFKFB1) in fish [165,166]. Overexpression of exogenous SREBP1a in the liver of *Sparus aurata* enhanced the expression of glycolytic enzymes GK and PFKFB1, decreased the activity of the gluconeogenic enzyme FBP1 and increased the mRNA levels of key enzymes in fatty acid synthesis, elongation and desaturation (acetyl-CoA carboxylase 1, acetyl-CoA carboxylase 2, elongation of very long chain fatty acids protein 5, fatty acid desaturase 2), as well as induced NADPH formation (glucose 6-phophate dehydrogenase) and cholesterol synthesis (3-hydroxy-3-methylglutaryl-coenzyme A reductase). As a result, chitosan-mediated SREBP1a overexpression caused a multigenic action that enabled the conversion of dietary carbohydrates into lipids (Figure 7), leading to increased circulating levels of triglycerides and cholesterol in carnivorous fish [157].

## 4. Conclusions

Characteristics such as nanoscale, low-toxicity, biodegradability, biocompatibility, derivatization, immunomodulatory effects, and easily affordable preparation conditions make chitosan a strong candidate for drug delivery into fish. Therefore, the use of chitosan in fish biotechnology has received growing attention in recent years. However, applications based on novel chitosan-based gene therapy methodologies to improve desirable traits in farmed fish have enormous potential for development. Most remarkable advances in the field addressed fish immunization, the control of reproduction for broodstock management and the modulation of gene expression to spare protein and overcome metabolic limitations of farmed fish. Further studies are needed for a better understanding of the extracellular and intracellular process, following chitosan-mediated gene delivery into fish. In addition, future trends in fish farming may greatly benefit from improved and more efficient chitosan formulations for enhancing gene delivery targeting and intracellular traffic in farmed fish.

## Figures and Tables

**Table 1:** Characteristics of chitosan–plasmid DNA polyplexes for studies performed in fish.

Preloading Diameter (nm)	Postloading Diameter (nm)	Preloading Zeta Potential (mV)	Postloading Zeta Potential (mV)	Encapsulation Efficiency	References
-	<10,000	-	-	94.5%	[145]
30–60	-	-	-	-	[146]
-	200	-	-	91.5%	[147]
-	-	-	-	83.6%	[148]
193 ± 53 ^1^	246 ± 74 ^1^	32.0 ± 1.0 ^1^	14.4 ± 1.3 ^1^	-	[80]
-	146 ± 2 ^2^	-	24.3 ± 0.5 ^2^	92.8% ± 1.4% ^2^	[149]
-	133	-	34.3	63%	[150]
-	50-200	-	-	97.5%	[151]
87	156	30.3	36.5	60%	[144]
-	743	-	-	98.6%	[152]
135	-	26.7	-	86%	[153]
-	-	-	-	84.2%	[154]
224 ± 62 ^1^	Similar to preloading diameter	33.0 ± 1.2 ^1^	14.4 ± 1.3 ^1^	-	[81]
-	750–950	-	-	98.6%	[155]
116	306	24.7	18.0	-	[156]
231 ± 18 ^2^	272 ± 36 ^2^	31.2 ± 1.5 ^2^	14.1 ± 2.3 ^2^	-	[157]
-	267	-	27.1	87.4%	[158]

^1^ Mean ± SD; ^2^ mean ± SEM.
